# A scoping review of the research evidence of the developmental assets model in Europe

**DOI:** 10.3389/fpsyg.2024.1407338

**Published:** 2024-07-02

**Authors:** Antonio David Martin-Barrado, Diego Gomez-Baya

**Affiliations:** Department of Social, Developmental and Educational Psychology, Universidad de Huelva, Huelva, Spain

**Keywords:** positive youth development, development assets, scoping review, Europe, wellbeing

## Abstract

**Introduction:**

Positive Youth Development (PYD) is a strength-based perspective that focuses on the resources to promote a successful transition to adulthood, derived from the Relational Developmental Systems theory. In this line, the Developmental Assets (DA) model focuses on describing the personal resources (Internal Assets) and contextual resources (External Assets) that help to reach PYD. Most research from this approach has been carried out in United States and, to a lesser extent, in the European continent. The aim of this scoping review was to examine the evidence collected for the DA model in Europe.

**Methods:**

Web of Science database was used to search for articles published between 2013 and February 2024. Initially, there were 55 possible articles to be included, but after applying the exclusion criteria, this number was reduced to 11.

**Results:**

The findings suggested that the evidence for DA in Europe was in line with North American research, so that a higher presence of DA was related to higher well-being, better psychological adjustment, and lower risk behaviors. Internal Assets were the most influential assets, with the Positive Identity category being especially remarkable. Commitment to Learning and Social Competencies were also highlighted in the studies included in the review. Concerning External Assets, Positive Boundaries-expectations and Empowerment were emphasized as protective factors in youth development. These assets were found to present a protective effect against risk behaviors and were positively associated with PYD and socioemotional skills.

**Discussion:**

Thus, these findings support the applicability of the DA framework in promoting PYD in European context, and some intervention strategies are discussed considering cultural diversity.

## 1 Introduction

Many researchers have argued that the transition from adolescence to adulthood has become longer than ever before, reaching up to the age of 29 (Sawyer et al., 2018; Wiium and Dimitrova, 2019). One of the developmental tasks facing young people during this transition is the search for their identity (Zacarés-González et al., 2009). First, a deficit model was adopted in the scientific literature on adolescence, which considered the adolescent as passive and a social problem to be managed (Hall, 1904; Brown and Prinstein, 2011). However, this negative conception only was effective to prevent maladaptive behaviors, and a complimentary positive approach was needed to foster positive outcomes (Lerner, 2004; Franco and Rodrigues, 2018; Lerner et al., 2021).

Relational Developmental Systems theory (Overton, 2014) is a meta-theory in developmental science which “emphasizes the relational structure of the individual as an active agent,

the centrality of the individual↔context relation, and a life span or life course developmental orientation” (p. 328). As indicated Overton (2013): “this meta-theory conceptualizes living organisms as active agents, that is, as relational, spontaneously active, complex adaptive systems, that are self-creating, self-organizing, and self-regulating” (p. 102). Relational Developmental Systems theory (Lerner et al., 2011, 2015) postulates that Positive Youth Development (PYD) occurs when there is an alignment between internal strengths (e.g., school commitment or positive future expectations) and contextual assets (e.g., adults who provide a safe environment). This development is characterized by a low presence of risk behaviors and a higher social contribution. PYD also focuses on the strengths of young people to facilitate a successful transition to adulthood, through the development of personal skills (Lerner et al., 2005; Geldhof et al., 2014). This approach is focused on the social engagement of youth and adolescents in education, family and community activities (Benson et al., 2006). In this line, this model states that every individual has the opportunity to present a positive and resilient development, regardless of their past experiences and adversities (Geldhof et al., 2014). Two models are integrated in this Relational Developmental Systems theory, which have been well-supported by literature: the 5Cs theory and the Developmental Assets model. Lerner et al. (2015) developed a PYD model with strong evidence, by describing five interrelated components or 5Cs: Competence (positive self-concept in different areas), Confidence (positive self-worth), Connection (positive relationships with others), Character (respect of cultural and social values) and Caring (sympathy and empathy to others).

Furthermore, Benson et al. (2004) introduced the Developmental Assets (DA) approach to describe the personal and contextual resources that promote PYD (Benson et al., 2011; Lerner et al., 2015). The approach was validated by Theokas et al. (2005). Table 1 shows a total of 40 assets divided into 20 Internal Assets and 20 External Assets, each one categorized into four distinct categories (Benson et al., 2011). The first group refers to the personal characteristics of adolescents, including Positive Values (e.g., Honesty and Equality), Commitment to Learning (e.g., Achievement motivation and School engagement), Social Competencies (e.g., Interpersonal and cultural competence) and Positive Identity (e.g., Self-esteem and Positive view of personal future). External Assets focus on social and contextual characteristics such as Support, Empowerment, Boundaries-expectations, and Constructive Use of Time. Support includes family communication, a supportive neighborhood, and a caring school climate. Empowerment refers to safe social environment and a community that values youth. Boundaries and expectations include positive school and family expectations and boundaries. Constructive Use of Time is related to engagement with creative activities and positive leisure time. In these eight categories, there are five distinct intervention contexts: personal, family, school, social, and community (Benson, 2007). This approach maintains a positive view by assuming that adolescents and youth are active and interactive with their developmental contexts (Benson et al., 2006). These assets are expected to be interactive and to reduce problem behaviors (Benson et al., 2011). These internal and external assets represent the dynamic process within adaptive developmental regulations across youth period. These developmental regulations between the individual and the context are adaptive when they are “beneficial to the maintenance of positive, healthy functioning of the components of a bidirectional relation (e.g., both individual and context, where the context can include, of course, other individuals)” (Lerner et al., 2016, p. 178).

**Table 1:** Description of the 40 development assets.

Developmental assets	
External assets	Internal assets
**Support**	**Commitment to learning**
1 Family support	21 Achievement motivation
2 Positive family communication	22 School engagement
3 Other adult relationships	23 Homework
4 Caring neighborhood	24 Bonding to school
5 Caring school climate	25 Reading to pleasure
6 Parent involvement in schooling	**Positive Values**
**Empowerment**	26 Caring
7 Community values youth	27 Equality and social justice
8 Youth as resources	28 Integrity
9 Service to others	29 Honesty
10 Safety	30 Responsibility
**Boundaries-expectations**	31 Restraint
11 Family boundaries	**Social Competencies**
12 School boundaries	32 Planning and decision making
13 Neighborhood boundaries	33 Interpersonal competence
14 Adult role models	34 Cultural competence
15 Positive peer influence	35 Resistance skills
16 High expectations	36 Peaceful conflict resolution
**Constructive Use of Time**	**Positive Identity**
17 Creative activities	37 Personal power
18 Youth programs	38 Self-esteem
19 Religious community	39 Sense of purpose
20 Time at house	40 Positive view of personal future

Based on Benson et al. (2004).

Benson (2007) found that community and society are two important sources that may generate assets for individuals’ development. The asset-generating community focuses on personal and community resources, while the asset-generating society refers to social norms, public policies, and values that promote assets’ nurture. Therefore, interventions to promote youth development and well-being can be implemented at both the microsystem level, such as parental behaviors, and at the macrosystem level, such as youth public policies.

### 1.1 Evidence of DA in the unites states

Some studies conducted with North American samples have supported the protective role of DA in promoting youth health and well-being, underlining their cumulative effect (Lenzi et al., 2015). Research suggested that an increased number of assets is associated with better school and work performance, as well as better well-being and decreased risk behaviors, such as alcohol or tobacco consumption (Scales et al., 2000; Murphey et al., 2004; Bleck and DeBate, 2016). These results were also observed in environments that would be classified as high-risk (Scales et al., 2017). A longitudinal study conducted by Scales et al. (2006a) found a positive prospective relationship between DA and academic achievement in the States (Scales et al., 2006a). High scores in school, family, and personal categories of DA have been also found to protect against suicidal ideation (Lensch et al., 2019). Researchers such as Pashak et al. (2018) observed in students in the Midwestern United States the protective effect of the presence of assets, regardless of whether they were Internal or External. The greater the number of these assets the lower mental health problems among students were detected. A research work, that gathered the participation of a sample of 25,000 young people from 31 countries aged 9 to 31 years, validated DA model as a valid approach for promoting PYD, regardless of their socioeconomic status, ethnicity, and culture (Scales et al., 2017).

However, many young people around the world have not developed the personal and contextual assets necessary for a successful transition into adulthood, despite the protective nature of these assets (Scales et al., 2016). Moreover, Scales et al. (2016) noted that the experience of DA tends to decrease during adolescence and youth, which may hinder a positive transition to adulthood. Some youth programs and community services may facilitate and develop asset categories such as Support, Boundaries-expectations, and Empowerment. Assets’ categories related with youth social engagement are usually fostered to prevent risk behaviors (Scales et al., 2006b).

Concerning the assessment, Developmental Assets Profile (Scales, 2011) is the most used instrument in the US and has received some international adaptations for countries around the world, such as, Albania, Bangladesh, Japan, Lebanon, and the Philippines. This 58-item self-report assesses the experience of the four internal and the four external assets previously described and has been linked to diverse indicators of psychological well-being in youth samples. Developmental Assets Profile presented good reliability and validity to work in diverse cultural settings.

### 1.2 Justification and aim

Adolescence is a critical life stage evidence to promote a healthy and adaptive transition is needed. Although there have been abundant research works conducted about DA framework in the United States, there is only few scientific articles in emerging adults in Europe (Benson et al., 2011). Therefore, additional evidence is required in Europe to gain validity of the contextual and personal resources to promote PYD. As well, more research is needed to provide reliability and validity of the instruments assessing DA in other contexts. Thus, the aim of this scoping review is to examine the quantitative evidence on the DA model in Europe. This evidence could have some practical implication to encourage youth programs designed from this framework.

## 2 Methods

### 2.1 Search strategy

A scoping review was used, instead of a systematic review, following the indications by Munn et al. (2018) and Arksey and O'malley (2005). A scoping review is a valid methodology because it is aimed at providing an exploratory in-depth coverage of available literature about a topic. This procedure is recommended to examine the extent, range and nature of research activity, to summarize and disseminate research findings and to identify research gaps in the existing literature. The aim of a scoping review is to address broader questions than a systematic review. In this scoping review, articles from 2013 to February 12, 2024, were selected from the international *Web of Science* database (Clarivate Analytics, London, United Kingdom). Initially, a total of 55 articles were identified for potential inclusion.

Subject, title, abstract, and indexing were used to search for the relevant articles. The search terms included the following terms and Boolean commands: “developmental asset*” AND youth OR “young people” OR adolescen* OR teenager* OR “emerging adult*” OR undergraduate* OR university OR “high school” OR student*.

### 2.2 Description of criteria for inclusion and exclusion

Criteria were established to select the articles. (a) The language criterion required that the articles should be written in English. (b) The participant criterion required that the participants belong to a country located at the European continent. (c) The articles with full-text availability, (d) The works may describe quantitative results, follow the DA model and administer an instrument to measure it (design criterion). (e) The publication period was established between 2013 and February 2024. (f, g) The article’s results and conclusions specifically analyze the DA (outcome criterion).

### 2.3 Data extraction

The present scoping review followed the indications of the Systematic Reviews and Meta-Analyses extension for Scoping Reviews (PRISMA-ScR; Urrútia and Bonfill, 2010; Tricco et al., 2018) to create an extraction form for the included articles. The indicators of these studies were title, abstract (structured summary), introduction (rationale, objectives), methods (protocol and registration, eligibility criteria, information sources, search, selection of sources, data charting process, data items, critical appraisal of individual sources, synthesis of results), results (selection of sources, characteristics of sources, critical appraisal, results of individual sources, syntheses of results), discussion (summary of evidence, limitations and conclusions), and funding.

Content analysis was performed by two psychologists with training in scoping reviews. This analysis was conducted in parallel and subsequently integrating the information collected. Methods and results were examined in detail in the articles in order to examine the quantitative evidence provided for the DA model in the specific European country. This information was organized into these sections: authors, aims, study design and sample, instrument, and main findings.

## 3 Results

### 3.1 Search results

Step 1. Search identification.

In the initial search, 55 articles were conducted in Europe between 2013 and February 2024 were found in the *Web of Science* database using Boolean commands and search terms.

Step 2. Initial analysis.

Following the previous approach, the next step involved analyzing the articles based on their titles and abstracts, considering the predetermined inclusion criteria. After this analysis, 34 articles were chosen for further analysis. 15 articles were excluded because of the sample, since they assessed samples from non-European countries. Additionally, three articles were eliminated because they were duplicates, two articles were excluded because they followed a qualitative methodology, and one article was not included because it described a literature review.

Step 3. In-depth analysis.

The full text analysis was conducted with the remaining 34 articles. Up to 18 studies were excluded because they did not follow a psychological approach and not use reliable and valid instruments to measure DA. Additionally, three studies were not included because they were not written in English, and two studies were non-empirical (they were narrative reviews in book chapters).

**Figure 1:**
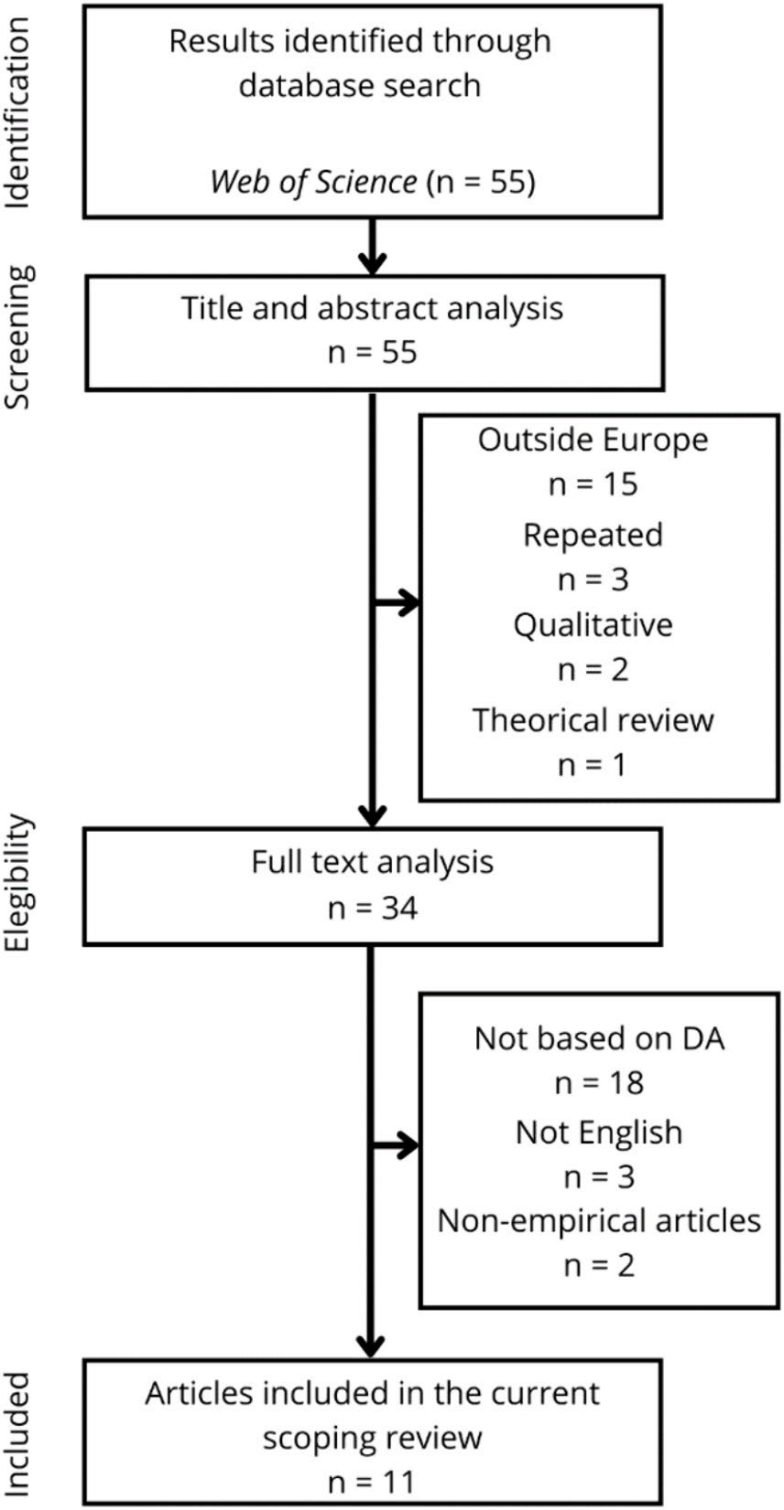
Flowchart of the scoping review.

Finally, there were 11 articles left that were considered relevant for the present scoping review. Figure 1 shows the steps followed in this review.

### 3.2 Instruments for DA assessment

The DA Profile instrument is the most used tool for measuring Developmental Assets (e.g., Search Institute, 2005, 2007, 2016). The questionnaire comprises 58 items that gather information on Internal and External Assets in various contexts, including individual, family, school, community, and society. Participants rate their experience of the assets on a 4-point Likert-type scale (1 – rarely, 2 – sometimes, 3 – often, 4 – almost always). This instrument showed high Cronbach’s alpha values.

Several studies used additional instruments to measure some variables to be correlated with DA. For instance, some works used the PYD-SF to assess the 5Cs of PYD (Geldhof et al., 2014), or the SWLS to measure life satisfaction (Diener et al., 1985). Other works used instruments to assess self-reported health (Ware et al., 1993) and mental health results.

### 3.3 Characteristics of the studies included

Table 2 shows the main characteristics of the included studies. All the articles included in this review followed a cross-sectional study design and employed a quantitative methodology. The total sample size of the 11 articles was 11,230 adolescents, aged between 13 and 29 years. Seven of the studies only included adolescent participants. Only four studies examined a wider time period, including participants aged 18 years to approximately 27–29 years. Concerning the proportion of male and female participants, most studies exhibited a bias in favor of females. Additionally, two studies did not report the age range of participants, and one did not report the Standard Deviation.

**Table 2:** Characteristics of the studies in the scoping review.

Author(s)	Aim(s)	Study design (Country and Sample)	Instrument(s)	Main findings
Dervishi et al. (2024).	To analyze the relationship between DA and life satisfaction among Albanian youth.	Cross-sectional. Albania. N = 409 (age 14–19, *M* = 18.68, no *SD* information provided; 58.9% were women).	DA: DA Profile (Search Institute, 2007). Life satisfaction: SWLS (Diener et al., 1985).	Few participants showed adequate levels of DA (Internal and External), including low levels of school, neighborhood, and adult support. There were no gender differences. Older age was associated with higher levels of DA, such as family support. Participation in recreational activities was very limited. The study found that individuals who had a greater presence of DA, particularly Internal ones (such as, Positive Identity, Positive Values, Social competence, and Commitment to Learning), reported higher levels of life satisfaction.
Dost-Gözkan et al. (2021).	To measure how Internal (Positive Identity) and External Assets (Support, Boundaries-expectations, and Empowerment) are experienced, as well as its associations.	Cross-sectional. N = 2055 (age 18–28; *M* = 20.41; *SD* = 2.04; 69.6% were women). Norway = 488; Romania = 255; Slovenia = 561; Turkey = 751.	DA: DA Profile (Search Institute, 2007).	External Assets were positively associated with a Positive Identity in the four countries, although Norway reported lower scores. DA was related to more well-being independently of the social environment. The family was a key context for DA in the four countries. Slovenian youth scored higher on External Assets. Norwegian and Slovenian youth scored higher in the Boundaries-expectations category. There was an inverse relationship between age and support, and a positive relationship between age and Empowerment.
Fernandes et al. (2021).	To provide an overview of DA and the 5Cs model.	Cross-sectional. N = 4,175 (age 15–25, *M* = 18.95, *SD* = 2.49; 62.5% were women). Kosovo = 900 (*M* = 16.34, *SD* = 0.97; 66.7% were women). Norway = 425 (*M* = 20.16, *SD* = 1.51; 73.5% were women). Portugal = 247 (*M* = 16.60, *SD* = 1.29; 57.9% were men). Slovenia = 648 (*M* = 19.81, *SD* = 2.63; 63.4% were women). Turkey = 974 (*M* = 19.96, *SD* = 2.46; 68.7% were women). *Ghana = 981 (*M* = 19.82, *SD* = 1.74; 52.5% were women).	DA: DA Profile (Benson, 2003). 5Cs: PYD-SF (Geldhof et al., 2014).	The prevalence of DA varied depending on the country’s conditions. Norwegian youth achieved the highest scores in Commitment to Learning, Social Competencies, and Empowerment. However, they scored lower in Positive Values. Slovenian youth showed high scores in Support and Empowerment. In Kosovo, there were low scores in Empowerment and Caring neighborhood. However, they scored high on Social Competencies and Positive Identity (especially Self-esteem). Turkish youth scored low on Boundaries-expectations and Constructive Use of Time, while scored high on Integrity.
Gomez-Baya et al. (2022a).	To analyze the relation between anxiety and DA, as well as gender differences. To explore whether gender differences in anxiety are explained by gender differences in DA.	Cross-sectional. Spain. N = 1,044 (age 18–28, *M* = 20.47, *SD* = 3.08; 75.5% women).	DA: DA Profile (Scales, 2011). Anxiety: Generalized Anxiety Disorser-7 (GAD-7; Spitzer et al., 2006).	Females scored higher on Boundaries-expectations and Support (External Assets), and Social Competence, Positive Values, and Commitment to Learning (Internal Assets). Males scored higher on Positive Identity. Empowerment and Positive Identity assets were negatively related to anxiety, while the Positive Values asset was positively associated. The study found that women had higher levels of anxiety when they scored low on Positive Identity and high on Positive Values.
Gomez-Baya et al. (2022b).	To analyze the association between DA and PYD in Spanish youth, as well as gender differences. To examine gender differences in PYD according to DA.	Cross-sectional. Spain. N = 768 (age 17–29, *M* = 19.50, *SD* = 2.27; 60.5% were women).	PYD: PYD-SF (Geldhof et al., 2014). DA: DA Profile (Scales, 2011)	The study found a positive relationship between DA and PYD, with a stronger correlation for Internal Assets. Gender did not affect the relationship. Men scored higher in Positive Identity, while women did in Empowerment, Support, Positive Values, Commitment to Learning, Social Competencies, and Boundaries-expectations. Men with higher Positive Identity were associated with higher perceived Competence, while women with high scores in Connection were associated with more Empowerment, Boundary-expectations, and Social Competencies. Women who scored higher on Caring also scored higher on Positive Values and Social Competence. Additionally, those who scored high on Character also scored high on Empowerment, Social Competencies, and Commitment to Learning.
Miconi et al. (2023).	To study the relationship between risk behaviors and DA in Egyptian and Roma Albanian youth.	Cross-sectional. Albania. N = 201 Egyptian and Roma Albanian adolescents (*M* = 16.63; *SD* = 1.80; 47% were women).	Risk behaviors: DA Profile (Search Institute, 2007). Well-being: WHO-5 (Dadfar et al., 2018) DA: DA Profile (Search Institute, 2007).	Positive Identity was the category with the lowest scores but more strongly related to well-being (especially in Egyptian youth). It was found that youth reported low family support and low neighborhood assets. Women reported more Positive Values, Social Competencies, and Family support. There were no differences in DA between those who attend school and those who do not. There was also no relationship between DA and risk behaviors.
Soares et al. (2019).	To explore whether DA are related to perceived life satisfaction.	Cross-sectional. Portugal. N = 503 (age 13–19; *M* = 15.92, *SD* = 1.17; 63% were women).	DA: Profile of Student Life: Attitudes and Behaviors (A&B) – Search Institute (2018) Life Satisfaction: SWLS (Diener et al., 1985).	Differences by gender, grade and age were observed. A higher presence of DA (mainly Internal) was linked to greater life satisfaction. The asset with the strongest effect was Self-esteem, followed by Sense of purpose, Planning and decision-making, School engagement, and Caring. However, the asset of Reading for pleasure was negatively associated. The External Assets that had the most significant impact on life satisfaction were Family support, Positive family communication, Support from other adults, and Youth as resources.
Soares et al. (2020a,b).	To analyze the relationship between DA and perceived health.	Cross-sectional. Portugal. N = 503 (age 13–19; *M* = 15.92, *SD* = 1.2; 63% were women).	Profile of Student Life: Attitudes and Behaviors (A&B) - Search Institute (2018) Perceived Health: SF-36 (Ware et al., 1993).	More DA were related to a more positive perception of health, especially Internal Assets (Self-esteem, Positive vision of personal future and Social Competencies). No gender or age differences were found. The External Assets with the strongest effects were Youth programs, Safety, and Youth as resources.
Vrdoljak et al. (2023).	To describe of DA and risk behaviors, as well as analyzing if DA can predict risk behaviors. To study gender differences.	Cross-sectional. Croatia. N = 728 (age 15–27, *M* = 18.41, *SD* = 2.29; 61.7% were women).	DA: DA Profile (Search Institute, 2016). Risk Behaviors: Items collected through several questionnaires.	High scores were observed in Internal Assets (Positive Identity, Positive Values and Commitment to Learning) and External Assets (Support, Empowerment and Boundaries-expectations). Low score was found in Constructive Use of Time asset. Higher scores in Social Competencies, Boundaries-expectations and Commitment to Learning were associated with fewer risk behaviors. Men reported more social competencies. The effects of DA were more protective in high school students than in undergraduates.
Wiium et al. (2019).	To assess DA by gender and parental educational level.	Cross-sectional. N = 1,234. Italy = 526 (*M =* 15.68, *SD* = 1.61; 41% were female). Norway = 592 (*M* = 16.71, *SD* = 0.91; 56% were female). Turkey = 116 (*M* = *15.68, SD = 1.09*; 63% were female).	DA: DA Profile (Search Institute, 2007).	High scores in DA were reported in the three countries, with Norway and Turkey experiencing more DA. Internal Assets were the most presented assets. More External Assets were found in Norway and Turkey, including Commitment to Learning and Support. Norwegian youth also reported higher Social Competencies, Boundaries-expectations, and Empowerment. Finally, Turkish youth have a more Positive Identity. Women in Norway reported more assets, including Support, Empowerment and Boundaries-expectations. Italian students who had fathers with a high level of education reported higher scores in Constructive Use of Time.
Wiium et al. (2021).	To analyze the relationship between DA and some indicators of poor mental health (sadness and suicide attempt), as well as analyzing the influence of the contexts.	Cross-sectional. Norway. N = 591 (age 15–19; *M* = 16.70, *SD* = 0.90; 55% were women).	DA: DA Profile (Search Institute, 2007). Poor mental health (Search Institute, 2016).	A negative relationship was found between DA and poor mental health. Higher scores in all DA categories (except Constructive Use of Time) were associated with less prolonged sadness. Empowerment, Positive Identity, and personal and family assets were protective against prolonged sadness. Family assets also had a negative effect on suicide attempts. Positive Identity had the greatest negative effect on prolonged sadness, controlling for the demographic variables.

*Country not included in this review because it is not a European country.

**Figure 2:**
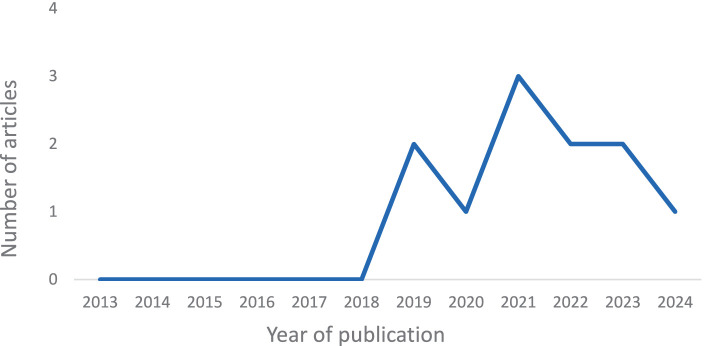
Distribution of articles by publication year.

The distribution of the publication year of the articles is shown in Figure 2. Despite the time was established in this scoping review from 2013 to February 2024, most articles come from 2021.

*Frontiers in Psychology* (5) was the journal that collected the largest number of articles. The following journals also published some articles on DA: *Frontiers in Psychiatry* (1), *International Journal of Adolescence and Youth* (1) *Ciencias Psicológicas* (1), *Child & Youth Care Forum* (1), *Applied Developmental Science* (1), and *Revista de Psicología* (1).

**Figure 3:**
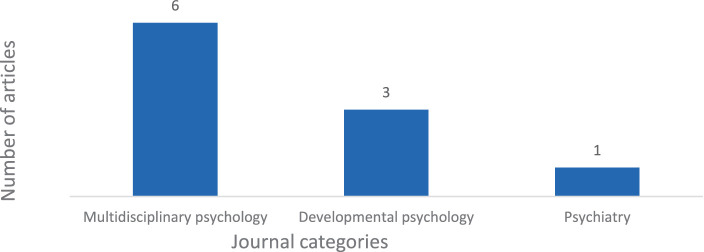
Distribution of the categories of the journals.

Figure 3 shows the categories distribution based on the Journal Citation Report (JCR) analysis. It is worth noting that one journal, *Ciencias Psicológicas*, was not included in any category or quartile since it was not included in the JCR during the publication year of the respective article.

**Figure 4:**
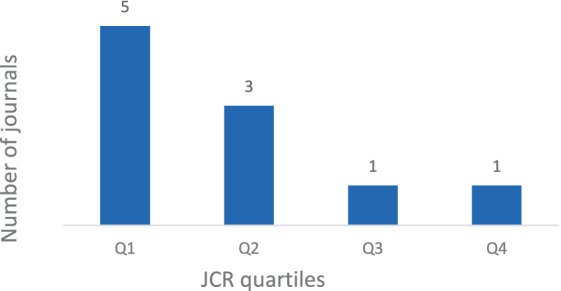
JCR impact factor quartile distribution of the journals included.

The JCR index also classifies the impact factor of the journal in quartiles, as shown in Figure 4. The journal *Frontiers in Psychology* reached the highest impact index with a score of 4.2 in Q1, while the lowest index was found in *Revista de Psicología* with a score of 0.4 in Q4.

Geographically, the 11 articles included in the study had samples from diverse locations across Europe. Some research even included multiple European countries, such as the study by Fernandes et al. (2021), which included adolescents from five European countries, or Dost-Gözkan et al. (2021), which incorporated four. The samples were collected from a total of 10 European countries. Most studies came from Norway (4), Portugal (3), and Turkey (3), while other works were developed in Slovenia (2), Spain (2), Albania (2), Romania (1), Croatia (1), Italy (1) and Kosovo (1). Thus, a very heterogeneous European sample was examined in the literature, from different regions of the continent.

### 3.4 Content analysis

All articles analyzed in this review are described in Table 2. All studies in the different European countries found that adolescents and youth with greater Internal and External Assets had more positive development. This positive development was characterized by greater well-being and lower presence of risk behaviors (e.g., Soares et al., 2019; Fernandes et al., 2021; Gomez-Baya et al., 2022a,b; Vrdoljak et al., 2023). Conversely, mental health problems were negatively associated with internal and external DA (Wiium et al., 2021). Several researchers have concluded that the Internal Assets presented stronger effect on psychological adjustment than the External ones (Wiium et al., 2019; Soares et al., 2020a,b; Gomez-Baya et al., 2022b; Dervishi et al., 2024). However, a study conducted with an Albanian sample found no relationship between DA and risky behaviors (Miconi et al., 2023).

After a deeper analysis to know which assets have been shown to be more relevant to specific outcomes, some results may be remarkable. The Self-esteem asset, in the Positive Identity category, has been shown to be the most important element among young people (Soares et al., 2019, 2020a,b; Vrdoljak et al., 2023). Its presence has been positively associated with External Assets (Dost-Gözkan et al., 2021) and has been found to be a protective element against anxiety (Gomez-Baya et al., 2022a) and prolonged sadness (Wiium et al., 2021). Several studies have found a positive association with well-being (Miconi et al., 2023), life satisfaction (Dervishi et al., 2024). and positive Boundary-expectations (External Asset; Dost-Gözkan et al., 2021). Additionally, Positive identity and Commitment to Learning and Social Competencies were found to be highly prevalent among Spanish (Gomez-Baya et al., 2022a,b), Norwegians (Wiium et al., 2019), and Croatian youth, where their presence was associated with less risk behaviors (Vrdoljak et al., 2023).

Empowerment scores were found to be elevated in Croatian (Vrdoljak et al., 2023), Norwegian (Wiium et al., 2019, 2021), Portuguese (Soares et al., 2019), Slovenian (Fernandes et al., 2021), and Spanish (Gomez-Baya et al., 2022b) youth. The lowest scores for the Boundary-expectations and Empowerment categories were observed in ethnic minority adolescents from Albania (Dervishi et al., 2024) while in adolescents from Kosovo the lowest score was observed in Empowerment (Fernandes et al., 2021).

Family context has been shown to be crucial in promoting positive developmental outcomes for adolescents, including protection against risky behaviors (Soares et al., 2019; Dost-Gözkan et al., 2021; Wiium et al., 2021; Dervishi et al., 2024). However, in certain countries, such as Albania, lower levels of well-being have been found, particularly among ethnic minority youth, such as Roma and Egyptians adolescents (Miconi et al., 2023). These groups have limited access to community, school, and family resources, and present lower Internal Assets. For instance, there were no notable distinctions between the youth who attended school and those who did not, except for the Social Competencies category, which reached a high score among those youth who were schooled.

The study conducted by Soares et al. (2019) found that DA varied by age, grade level, and gender. In this line, Vrdoljak et al. (2023) concluded that there were more DA in youth enrolled at high school students than in undergraduates. The Empowerment category has shown a positive relationship with age, in the sense that the older the age, the higher the score (Dost-Gözkan et al., 2021). Furthermore, gender differences were observed in the presence of assets, with males scoring higher on Positive Identity, which was associated with the Competence component of the 5Cs. According to Gomez-Baya et al. (2022a), women scored higher on Empowerment, Support, Positive Values, Commitment to Learning, Social Competencies, and Boundaries-expectations. These higher scores were associated with better scores in the Connection, Compassion, and Character components. The category of Constructive Use of Time has proven to be a controversial element in some studies. This asset had the lowest scores among participants in several countries (Fernandes et al., 2021; Vrdoljak et al., 2023). Only the study by Wiium et al. (2019) with Italian students found a correlation between high scores in Constructive Use of Time and a high educational level of fathers.

## 4 Discussion

Past literature in Psychology described correct youth development as the absence of risk behaviors, such as alcohol or drug consumption or risky sexual practices (Lerner et al., 2005; Brown and Prinstein, 2011). However, in recent decades, PYD approach emerged, which focused on the strengths of young people (Lerner et al., 2011; Geldhof et al., 2014). From this perspective framework, there is a model focused on the personal and contextual assets, called DA model (Benson et al., 2006). This approach has gathered significant scientific evidence, mainly in the United States (e.g., Theokas et al., 2005; Scales et al., 2006a). The aim of this scoping review was to determine whether the evidence from the DA model is applicable to Europe. As far as we know, this is the first scoping review to reach this aim.

This scoping review was conducted by including articles from 2013 to February 2024 indexed in the *Web of Science*. Although55 articles were initially included, after applying the exclusion criteria, a total of 11 articles were selected to integrate the present work. Regarding bibliometric criteria, almost all the articles belong to the field of Psychology (including Multidisciplinary, Developmental psychology, and Psychiatry). Regarding the sample’s origin, several European countries were included, with Norway, Portugal and Turkey being the most frequent ones.

The DA model proposed by Benson et al. (2004) has been extensively studied since the beginning of this century, mainly with US samples (e.g., Scales, 2011; Scales et al., 2017; Lensch et al., 2019). DA framework is based on decades of research and is already in extensive use in the US and some other countries as well, as showed the validations in Albania, Bangladesh, Japan, Lebanon, and the Philippines by Scales (2011). However, in this review, even covering a wide time period from 2013 to early 2024, the first study included was from 2019. Despite the extensive scientific research conducted in North America, the model has only recently gained scientific interest in Europe. This may be due to the cultural, linguistic, and geographical diversity of the continent, as well as a difficulty to adopt models from other culture and a different language. The majority of articles on this topic have been published within the last 4 years. Therefore, there appears to be a growing interest in researching DA. Furthermore, the articles have been published in several high-impact journals, particularly those ranked in Q1 and Q2 of JCR index.

The articles in this scoping review provided evidence for the benefits of DA in Europe. Youth who scored higher on the DA showed better psychological adjustment, greater well-being, higher life satisfaction, better socioemotional skills, and fewer risk behaviors (e.g., Dost-Gözkan et al., 2021; Gomez-Baya et al., 2022b; Vrdoljak et al., 2023). Low scores on the DA were found to be associated with more psychological problems (Wiium et al., 2021) and more barriers to positive development (Miconi et al., 2023). These findings are consistent with U.S. research, which has shown that a high DA score is related to more PYD (Scales et al., 2000; Murphey et al., 2004; Benson et al., 2011; Pashak et al., 2018).

The model of the DA distinguishes between Internal and External Assets. The Internal assets had stronger effects on psychological outcomes among young people in the included studies (e.g., Soares et al., 2020a,b; Gomez-Baya et al., 2022b). The categories of Positive Identity, Social Competencies, and Commitment to Learning were the most remarkable assets according to the results examined.

First, the asset of Self-esteem, categorized as Positive Identity, has been identified as one of the most prevalent and protective assets for young people (e.g., Soares et al., 2019, 2020a,b; Wiium et al., 2021; Gomez-Baya et al., 2022a; Vrdoljak et al., 2023). This may be due to the significance that adolescents attribute to the search for self-identity (Zacarés-González et al., 2009). The development of a positive and stable self-image may be associated to greater well-being, achievement, and better Social Competencies (Greenberg, 2008). Positive Identity reflects the perceived control over one’s life and positive feelings about oneself and the future. These elements are related to healthy development (Scales et al., 2016).

Second, the importance of Social Competencies was very underscored in some studies (Wiium et al., 2019; Gomez-Baya et al., 2022a,b), being associated with fewer risk behaviors in adolescents (Vrdoljak et al., 2023). High scores on assets such as Peaceful conflict resolution, Resistance skills or having the ability to Planning and decision-making were related to better positive development (Masten et al., 2006; Burt et al., 2008). In countries such as Albania, young people who went to school scored higher on Social Competencies (Miconi et al., 2023) than those who did not. This finding may imply that even schools in at-risk environments can positively influence the social–emotional skills.

Third, several works (Wiium et al., 2019; Gomez-Baya et al., 2022a,b; Vrdoljak et al., 2023) found important scores in Commitment to Learning. This category is related to high levels of intrinsic motivation among young people, which can drive learning and positive development. According to Larson and Rusk (2011), young people may possess the ability to generate creative and effective solutions to problems they encounter, as well as demonstrate greater perseverance in activities and deeper information processing. De Carvalho and Schumacker’s (2012) study also highlighted the importance of the Commitment to Learning category, along with Boundaries-expectations, in preventing juvenile delinquency and reducing risk factors.

The categories of Empowerment and positive Boundaries-expectations were prominent in relation to External Assets. Some studies from diverse countries have underline the importance of empowerment (Soares et al., 2019; Wiium et al., 2019, 2021; Fernandes et al., 2021; Gomez-Baya et al., 2022b; Vrdoljak et al., 2023). This concept suggests that young people play active role in their environment, which can lead to a sense of security and foster both perceived competence and personal autonomy. Moreover, several researchers have demonstrated that this category provides very developmental benefits (Scales et al., 2006b; Lee et al., 2007; Scales, 2011).

Regarding Boundaries-expectations, some studies have concluded the importance for youth development. Appropriate supervision by parents (or the school) can provide a model to follow based on established rules. This can help young people become more autonomous, gaining more security, and increased trust and bonding with them, which in turn may establish the necessary conditions to develop a Positive Identity. However, excessive control may lead to psychological problems (Lansford et al., 2014). Similarly, a high score in positive expectations has been linked to positive psychological adjustment and less risk behaviors (Scales et al., 2006b; Kobak et al., 2017). Research has shown that a good family climate and organization is relevant for the promotion of DA and can lead skills’ development (Ryan and Deci, 2017). Moreover, this effect may moderate some vulnerabilities that limit the experience of DA (Soares et al., 2019; Miconi et al., 2023; Dervishi et al., 2024) and reduce the decrease in DA found in older ages (Scales et al., 2016), as found in the study by Vrdoljak et al. (2023). Assets’ development in family and community may help youth to reach an adaptive transition thanks to their role as asset generators (Benson, 2007).

Constructive Use of Time was a category in which participants scored lower (compared to the other assets) in several studies (Fernandes et al., 2021; Vrdoljak et al., 2023). This finding was unsuspected, since studies have shown positive effects of community-oriented leisure activities involving socialization (Di Bona, 2000; Auhuber et al., 2019). However, having too many activities can lead to oversaturation and diminish the pleasure derived from them. Leversen et al. (2012) discovered that life satisfaction is positively associated with involvement in leisure activities, as long as they are meaningful to the adolescent and satisfy their needs. It should also be noted that this category has a low reliability within the DA Profile scale.

Finally, studies assessing youth from Kosovo (Fernandes et al., 2021) and Albania obtained some worth noting results (Miconi et al., 2023; Dervishi et al., 2024). Kosovar youth obtained low scores in Commitment to Learning and Empowerment. Kosovo is a young state with high poverty rate in which youth population faces difficulties in employment access (Bellaqa, 2021). In Albania, no relationship was found between DA and risk behaviors (Miconi et al., 2023). This may be due to the marginalization, insecurity and violence experienced by young people. Additionally, the COVID-19 pandemic may have exacerbated this inequality (Miconi et al., 2023). Additional public policies are necessary to ensure safety in school and community and promote social justice.

These results observed in European samples are consistent with those observed in US samples (Scales et al., 2000, 2006a,b; Scales, 2011), providing a cross-cultural validation of the DA model to explain positive psychological adjustment in European youth samples. The results included in the present scoping review underline the importance of the Relational Developmental Systems model (Overton, 2014) to understand youth development within an interactive process between the individual skills and the contextual resources. This model underlines the resources which should be fostered in the contexts, but also highlights the role of young people as active agents in their own development and the importance of their contribution to improve their contexts. The results observed in the European studies concluded that both internal and external resources are needed within a dynamic and interactive process in the different cultural frameworks in the European continent.

### 4.1 Strengths and limitations

Some limitations of this scoping review should be acknowledged. First, the exclusion criteria for language (only English articles) and type of scientific paper (only journal articles) may have resulted in the exclusion of papers that could have been of interest. As well, only publications included in the Web of Science have been included. Despite the importance of this database, future reviews about this topic could also use other databases. Additionally, the authors of this review acknowledged the potential for personal bias that may affect the analysis of the results. Second, more than a half of the articles included in this review were published after the COVID-19 pandemic, which may have influenced young people’s perception of their strengths. Third, all the articles included in this study followed a cross-sectional design, which means that the conclusions are based on bivariate associations between variables without power for predictive or causal inferences. Fourth, most of the articles included had sample sizes of less than 1,000 participants; only four articles had sample sizes between 1,000 and 4,000 adolescents. Fifth, it is important to indicate that most samples were chosen for convenience, mainly from student samples. Another limitation of the studies is related to the age range, as many studies focused on early, middle, and late adolescents (ages 10–18), with few based on emerging adult samples (ages 18–29). This difference between age groups may difficult the results’ generalization. Moreover, the samples of the studies were predominantly composed of females, what may bias the results and limit the applicability. Thus, the generalization and applicability of the results may be improved by sampling procedures controlling for gender and age representation, as well as including youth population not enrolled at universities and high schools.

Despite these limitations, the present review has some notable strengths. First, a heterogeneous sample has been gathered from various countries in Europe. Secondly, a total sample of 11,230 young people between 13 and 29 years of age was examined. Third, most studies used the same instrument to measure DA, the DA Profile scale (e.g., Search Institute, 2007, 2016). This scale presented high levels of internal consistency. Finally, this scoping review has provided a comprehensive review of the European studies on DA, with a balanced focus on internal and external assets supporting PYD.

### 4.2 Implications for research, policy and practice

More research is needed to found validity of DA model in the European continent, since evidence has been collected only in few countries. For example, no samples were found from other countries such as Germany or France. Further research is needed in these and other countries to examine whether the DA can be generalized to these contexts. Longitudinal designs are recommended to analyze the directionality in the relationships between variables, and randomly collected samples, also including young people outside the educational context, are necessary to generalize results. A greater number of studies with emerging adults are recommended. Additionally, it is recommended to include larger sample sizes and ensure equal representation of genders to avoid potential biases in the results.

Although both the 5Cs and DA models defend a positive perspective of adolescent transition, the former has been studied more extensively. A recent scoping review by Martin-Barrado and Gomez-Baya (2024) analyzed the evidence for the 5Cs model and found consistent results in European and North American contexts. Similarly, the DA have been found to be similarly protective and beneficial in Europe and North America, although more evidence is still needed in Europe. Future research should focus on integrating the 5Cs model and DA into the Relational Developmental Systems theory (Lerner et al., 2011), as pointed out the study by Gomez-Baya et al. (2022b).

Furthermore, although the instrument used by most studies (DA Profile) to assess DA has demonstrated good validity and reliability, future studies should consider adopting a mixed methodology, by integrating qualitative and quantitative methodologies. This approach can reveal relationships and implications that a purely quantitative approach may not appreciate. To date, only two qualitative works have been developed following DA model. Miconi et al. (2021) conducted six focus groups with a sample of Egyptian and Roma adolescents in Albania. These authors concluded that the participants reported a low level of DA and important barriers to access to them, concerns about mental health and coping, the influences of some experiences of discrimination, integration and society contribution, and the importance of proximal developmental contexts. Furthermore, Uka et al. (2022) collected qualitative evidence for the role of DA in the effectiveness of Internal Cohesion Psychotherapy in treating a sample of 10 youth with depression and anxiety in Kosovo. These authors performed semi-structured interviews and concluded that DA could be integrated in the psychotherapy to enhance the effectiveness, underlining the importance of both internal and external assets. Further evidence is needed to integrate qualitative and quantitative evidence of DA model in Europe.

The results of this review could be significant because they may have some implications for practice. This scoping review found a negative correlation between DA and risk behaviors, as well as concerning anxious and depressive symptomatology. Therefore, interventions aimed at promoting health and preventing these problems in psychological adjustment should focus on enhancing the competencies and resources of young people based on DA. Programs should include measures to promote the Positive Identity, including Personal power, Self-esteem, Sense of purpose, and a Positive vision of personal future. Other important assets include Boundaries-Expectations, Commitment to Learning, Social Competencies, and Empowerment. Strategies to develop these assets can be useful in reducing and preventing certain mental health problems, such as anxiety (Gomez-Baya et al., 2022a). Similarly, research has highlighted the importance of the family to nurture assets for a positive development. As in the US, in European countries, families are a remarkable source of support and positive attitudes to live in society (Scales et al., 2016). Therefore, including families in promotion and prevention programs of DA can lead to stronger outcomes.

In terms of public policy, governments should facilitate opportunities for young people to participate in environments that promote a correct PYD (e.g., volunteering and leisure active activities). Studies have shown that activities involving socialization are particularly satisfying (Di Bona, 2000). In the context of mental health and risk behaviors, health and educational institutions should move away from the traditional medical model and adopt a psychosocial approach. This includes providing resources, support, and activities that promote positive development and prepare young people for the challenges of adulthood (Scales et al., 2016), such as university tasks, work or independence. Regarding educational context, adolescents and youth spend a significant portion of their lives in school, high school and university, what indicated that educational environment is an excellent context to foster necessary personal skills. In the United States, the Search Institute (2024) has developed several Out-of-School Time (OST) youth programs aimed to develop social–emotional skills and enhance leadership abilities through sports, tutoring, arts, or school support. These workshops should be implemented for European youth, considering the cultural differences of each country. Based on Relational Developmental Systems Theory, youth agency should be fostered to be able to create adaptive regulations within their developmental contexts. Based on the respective cultural norms and characteristics, young people may discover and write their own life projects aimed to provide a positive contribution to society and reach positive developmental outcomes at an individual level, such as well-being, healthy lifestyles, or educational and labor success. Programs to enhance DA should take into account the plasticity of youth development across life span and the specific ways in which DA could be promoted in each cultural context across Europe. Because the relational nature of youth↔context development, specific characteristics should be previously analyzed for program design, by analyzing all the sociocultural aspects of the communities. At this regard, although DA model has reached some validity in diverse European countries, the diversity of youth and context realities in these countries should be accounted for implementing effective initiatives. Europe’s diverse cultural landscape could affect the development and impact of DA, controlling for the socio-economic, educational, and community variables.

## 5 Conclusion

The aim of this scoping review was to examine the evidence for DA model (Benson et al., 2004) in Europe. The results found in Europe are consistent with previous research conducted with U.S. samples. Higher scores in the DA (both internal and external assets) were associated with better psychological adjustment, improved academic performance, greater socioemotional skills and reduced risk behaviors. Additionally, results showed that DA instrument is a reliable and valid tool for assessing the capabilities and resources of young people to reach PYD. Importantly, Internal Assets appeared to have a greater influence, and Positive Identity category was found to be the most salient one. Despite linguistic, cultural and socioeconomic differences in the European countries, Commitment to Learning, Social Competencies, Empowerment, and Boundaries-expectations were prominent assets.

Finally, some public policies should be designed to improve the well-being of young people from educational and community contexts and foster job opportunities. Additionally, interventions aimed at promoting positive development should involve the family and the neighborhood to achieve a greater impact. DA-based training to cope with future challenges in adulthood can promote a resilience in this developmental transition. Underlining the importance of youth agency, public policies should foster youth empowerment to create adaptive regulations within their own contexts. DA model may be a valid framework to guide program design to foster PYD and social contribution in Europe, as already pointed out in the US. Diversity of cultural backgrounds in Europe should be addressed to design effective programs.

## Data availability statement

The original contributions presented in the study are included in the article/supplementary material, further inquiries can be directed to the corresponding author.

## Author contributions

AM-B: Conceptualization, Formal analysis, Investigation, Methodology, Writing – original draft, Writing – review & editing. DG-B: Conceptualization, Funding acquisition, Resources, Supervision, Writing – original draft, Writing – review & editing.
